# How users make online privacy decisions in work and personal contexts of use

**DOI:** 10.1038/s41598-024-70718-7

**Published:** 2024-08-27

**Authors:** Jennifer Klütsch, Christian Böffel, Lukas Abraham, Janina Mooser, Dominik Thüs, Parwana Tawanger, Sophia von Salm-Hoogstraeten, Sabine J. Schlittmeier

**Affiliations:** 1 Institute of Psychology, Work and Engineering Psychology, RWTH Aachen University, Jägerstraße 17-19, 52066 Aachen, North-Rhine Westphalia Germany; 2 Empirical Educational Research, Saarland University, Saarbrücken, Germany; 3 Saint-Gobain Research, Herzogenrath, Germany

**Keywords:** Context of use, Trust, Privacy decision-making, Remote work, Digital competence, Human behaviour, Psychology

## Abstract

With the rising usage of contactless work options since COVID-19, users increasingly share their personal data in digital tools at work. Using an experimental online vignette study (*N* = 93), we examined users’ willingness to use a video conferencing tool, while systematically varying the context of use (personal vs. low trustworthiness work vs. high trustworthiness work) and the type of information shared (low vs. medium vs. high sensitivity). We also assessed users’ perceived responsibility in work and personal contexts of use and their self-assessed digital competence. Our results highlight employer trustworthiness as an important factor in the willingness to use a third-party video conferencing tool, with increased willingness to use these tools in work contexts of use with high trustworthiness compared to those with low trustworthiness. This effect seems to be reduced when the data to be shared is of high sensitivity, compared to medium and low sensitivity data. Furthermore, despite reduced responsibility for data protection in work compared to personal contexts of use, the willingness to use a video conferencing tool did not decrease between trustworthy work and personal contexts of use. We discuss our findings and their methodological implications for future research and derive implications for privacy decisions at work.

## Introduction

The COVID-19 pandemic rushed the implementation and use of videoconferencing tools^1^ and increased their demand. Consequently, videoconferencing tools became essential in many people’s lives^2^. However, the use of such virtual tools is accompanied by many online privacy choices, even in the work context^1,3^. Consider an employee who uses a videoconferencing tool such as Zoom for weekly team meetings. Before the first meeting, the video conferencing tool requests access to the microphone, camera, and calendar, and offers to link itself to his/her professional networking profile. While the microphone and camera access are inevitable, access to the calendar or a networking profile could collect additional data. Consequently, the use of such third-party videoconferencing tools exposes the employee to privacy risks where he/she becomes responsible for his/her data protection not only as a private individual but also as an employee. Given the contextual nature of privacy^4^, the adoption of virtual tools could lead employees to dismiss privacy violations. This might occur as the employees might base their privacy decision on the employer’s trustworthiness or shift the responsibility for data protection to their employers when deciding whether to use digital solutions at work. As a consequence, employees could engage in more generous privacy decisions with operators of videoconferencing tools at the work context of use. Nevertheless, little is known about whether these privacy decisions stem from trust in employers or a shift in perceived responsibility to the employer. We address this research gap and identify how employer trustworthiness and shifts in responsibility for one’s own data protection to the employer affect the willingness to use a third-party video conferencing tool in work contexts of use compared to personal contexts of use. In the following, we summarize the theoretical background to these research questions.

### Biased privacy decisions

Users’ decisions to disclose personal data to digital services, often referred to as informational privacy^5,6^, are defined as their control over “when, how, and to what extent information about them is communicated to others” (p. 5)^7^. Current privacy research highlights how users base these privacy decisions and the resulting data disclosures on cognitive heuristics, biases or other influences, such as trust in the data recipient^8–12^. Rather than acting as purely rational agents who weigh up potential risks and benefits associated with a privacy decision (cf. privacy calculus theory^13^), users also seem to base these decisions on aspects which are not necessarily related to them. For instance, it has been shown that privacy decisions are influenced by misleading design cues, so-called *dark patterns*. Dark patterns “[trick] users into performing unintended and unwanted actions, based on a misleading interface design” (p. 239)^14^. For example, users are influenced by the default option, such as cookie options being preselected^15^. Besides these more or less visible design aspects within technological applications, privacy decisions seem to be highly contextual^4,6,11,16^. Even though the context of a privacy decision is often examined through investigating "where" information is disclosed online, such as on websites^16,17^, during app downloads^18–20^ or on social networking sites^21,22^, the contextuality of privacy decisions refer to a broader concept. Masur^6^, for example, defines context as “a stable set of characteristics, which in their own way shape our perceptions of privacy” (p. 144)^6^. According to Nissenbaum^4^, contextual characteristics can be categorized by five parameters: (1) the sender, (2) the subject and (3) the recipient of information, (4) the information type and (5) the transmission principles referring to the constraints under which information is disclosed. According to the theory of contextual integrity^4^, these characteristics define whether users perceive their privacy as protected or not and, thus, their personal data disclosure as appropriate. The theory offers valuable insight into how the context affects users’ privacy decisions and their perception of privacy violations. Researchers support the theory of contextual integrity^23–25^. For example, studies show that acceptance of data disclosures to smart speakers decreases under certain transmission principles^23^. Similar effects were examined by Kokolakis^11^ who highlighted differences in familiar contexts compared to commercial contexts. Comparing study results from Norberg et al.^26^ and Tsai et al.^27^, the researcher highlights that in familiar contexts, participants provided more data than originally intended (non-protective privacy behavior) whereas in commercial contexts, participants were willing to pay for premium service to secure their privacy (protective privacy behavior)^11^. Other researchers underscore the importance of the data recipient. For instance, studies show that users are less willing to disclose personal data on financially sensitive websites (like banking sites) than on less sensitive ones (like travel sites)^17^. The contextual nature of privacy thus suggests that users may also differ their privacy decisions based upon the context of use. Consequently, the shift from the personal to the work context of use could lead them to perceive less privacy violations, even though the data recipient remains the same. One reason may be that employees base their privacy decisions whether to share data with a third-party remote work tool (e.g., video conferencing tool) on the trustworthiness of the employer.

### The role of trust

Previous research has shown that trust can be an important determinant for users’ willingness to disclose personal data to technological applications (e.g., websites, apps)^10,16,28^. Trust can be defined as a “psychological state comprising the intention to accept vulnerability based upon positive expectations of the intentions or behavior of another” (p. 395)^29^ and is based upon three major trustor characteristics: (1) integrity, (2) ability, and (3) benevolence^30^. Actions that increase trustworthiness of an employer are, for example, (a) employee integration into the ongoing work process, (b) personnel responsibility by personnel development measures or occupational health and safety, (c) mutual support in self-coordination by appropriate organizational structures or leadership and/or (d) employee participation in reorganization or optimization^31^.

Within privacy research, trust is found to be an antecedent and outcome of privacy, and it is a mediating variable as well as a moderator of privacy concerns and behavior^16^. Additionally, Gerber et al.^28^ found trust to be a good predictor for privacy attitude and behavioral intention. To identify impacts of trust on users’ data disclosure, trust is often measured for the data recipient such as the company behind a technological application^32–34^ or experimentally manipulated for the technological application, e.g., through its appearance^35^ in privacy research. For instance, Schoenbachler and Gordon^34^ examined how trust in a company reduces concerns about privacy and increases consumers’ willingness to provide personal data to the company itself. Additionally, the brand of online providers has been found to be an important trust cue for users^36–38^. Other researchers examined employees to depend their data disclosures at work (e.g., for correspondence or on their performance) on strong trust beliefs with lawful data processing by the employer^39^. Yet, it remains unclear whether employer trustworthiness can act as a determinant for users’ privacy decisions to third-party video conferencing tools, even if the employer does not collect and manage the data themselves. It could be argued that the employer’s recommendation to use the video conferencing tool leads to generous privacy decisions, because a trustworthy employer is expected to act in one’s interest^30^. On the other hand, it could be that the trustworthiness of the employer is transferred to the trustworthiness of the data recipient and serves as an anchor (see anchor heuristic^40^). We therefore investigate employer trustworthiness as a determinant of data disclosures to third-party video conferencing tools in a work context of use.

### Shifted responsibility in work contexts of use

Within work contexts of use, the responsibility for data protection could also be shifted to the employer. ‘Perceived responsibility’ is often investigated in psychological research, for example in relation to the ‘responsibility diffusion effect’. This term describes the phenomenon whereby individuals feel less responsible for their actions when they believe that others are also responsible^41^. Within work contexts of use, employees are often directed to perform certain actions at work, and might even be ordered to use a certain software or tool by the employer. This could also cause employees to feel reduced responsibility for their data protection in work contexts of use. Therefore, it can be assumed that the responsibility for data protection could also be shifted to the employer. This could then lead to higher willingness to share personal data and to use video conferencing tools, even if the same data recipient requires the same type of information as in a personal context.

### From low to high sensitivity of information

Highly sensitive information could reduce these effects of employers’ trustworthiness and responsibility. Differences in users’ privacy decisions regarding different types of information are extensively documented in privacy research^25,42,43^ and highlighted as an important parameter for users to evaluate the appropriateness of their data disclosure^4^. For example, it has been shown that sharing demographic data is perceived as less aversive than sharing location data or browsing history^42^ whereas information such as phone number is perceived as particularly sensitive^44^. Hence, if users perceive certain types of information as off-limits, the impact of trustworthiness and shifted responsibility in work contexts of use could be limited as users may protect highly sensitive types of information more regardless of context. Another reason why we propose to investigate the context of use, and its interdependence with different types of sensitive information, is because context changes how information is perceived^25^. For example, Martin and Nissenbaum^25^ demonstrated that types of information (e.g., on friends, location, politics) can be viewed differently depending on the context in which the information is collected. As an example, the researchers explain that sharing sensitive information (e.g., purchase information) can be considered appropriate in contexts where its collection is expected (e.g., retail contexts) but inappropriate in contexts where its collection is not expected (e.g., health insurance contexts)^25^.

### Users’ digital competence

Previous literature has examined privacy literacy as an important antecedent of users’ privacy behavior. Privacy literacy refers to users’ knowledge about the technical, legal and institutional aspects of protecting their data on the Internet as well as their ability to regulate and protect their data^45,46^. Privacy literacy has been found to improve users’ privacy decisions^45,47^. For example, participants with high privacy literacy were more likely to engage in information control behaviors such as clearing their browser history or hiding their real name^47^. In addition, it has been found that participants with high privacy literacy exhibited more cautious privacy behaviors and higher levels of perceived privacy security in social network systems such as Facebook^45^. However, researchers such as Büchi and co-authors^48^ also highlight the importance of general Internet skills, which have been found to be associated with improved user control over information and can reduce users’ exposure to privacy risks. To support users’ privacy decisions online, the researchers therefore emphasize the “need for adaptive public policies that also ensure that universal and transferable digital skills are constantly developed, maintained, and enhanced in the light of continuous media change.” (p. 20)^48^. One approach to this is presented by the European Union and lies in improving digital competence, which can be referred to as the general ability to use digital media and the following key areas of competence: (1) seek & process, (2) communicate & collaborate, (3) produce & present, (4) protect & act safely, (5) problem-solving & usage and (6) analyze & reflect^49–51^. Digital competence is identified by the European Union as a key component for the self-dependent and self-critical use of technological applications within work, leisure, or communication contexts^52^. In addition, digital competence is associated with the ability “to retrieve, assess, store, produce, present and exchange information, and to communicate and participate in collaborative networks via the Internet” (p.15)^52^. The results on general Internet skills^48^ emphasize that whether users are influenced by the potential effects in work contexts of use, such as shifted responsibility, could be related to their level of digital competence. We thus expect that participants with higher self-assessed digital competence (a) are less willing to share data in personal and work contexts and (b) are less likely to be influenced by employers’ trustworthiness or by shifted responsibility in their privacy decisions at work.

### Present study and hypothesis

The overarching goal of this study is to understand how users make online privacy decisions in work contexts of use. Therefore, we systematically vary perceived trustworthiness of the employer and investigate whether employer trustworthiness affects users’ willingness to use a third-party video conferencing tool in work contexts of use. We expect that willingness to use will be higher when employers are trustworthy than when they are not trustworthy in work contexts of use (Hypothesis 1) because employer trustworthiness might serve as an anchor for the trustworthiness of the data recipient^40^. Additionally, we systematically varied the personal context of use in comparison to the work context of use and captured perceived responsibility for data protection in work compared to personal contexts of use. Because users might shift responsibility for their data protection to trustworthy employers at work, we expect that the willingness to use a videoconferencing tool will be higher in trustworthy work contexts compared to personal contexts of use (Hypothesis 2). As effects of both trustworthiness and responsibility could depend on the type of information in question, we vary the sensitivity of information types to be released when declaring consent. We expect reduced willingness to use a video conferencing tool when more sensitive information types are requested, for instance, browsing history in comparison to demographic data (Hypothesis 3). Finally, it is tested whether individual differences in participants’ self-assessed digital competence affect privacy decisions. We expect that higher self-assessed digital competence reduces the willingness to use a video conferencing tool (Hypothesis 4.1). Moreover, we expect that self-assessed digital competence reduces any potential increase in willingness to use a video conferencing tool for high trustworthiness work contexts of use in comparison to low trustworthiness work or personal contexts of use (Hypothesis 4.2).

## Methods

In an experimental online vignette study, participants were instructed to imagine themselves in certain vignette scenarios and asked to indicate how willing they would be to use the described video conferencing tool. The vignette scenarios varied within subjects in the *context of use* (personal vs. low trustworthiness work vs. high trustworthiness work) and the *type of information* (low sensitivity vs. medium sensitivity vs. high sensitivity) shared with the video conferencing tool. Self-assessed digital competence served as a covariate, while the measurement of perceived trust in the described (un-) trustworthy employers served as a treatment check. Additionally, perceived responsibility for data protection in work and personal contexts of use and reasons for (not) using the video conferencing tool were assessed. The study was conducted with the questionnaire tool Lime Survey^53^.

### Procedure

**Fig. 1:**
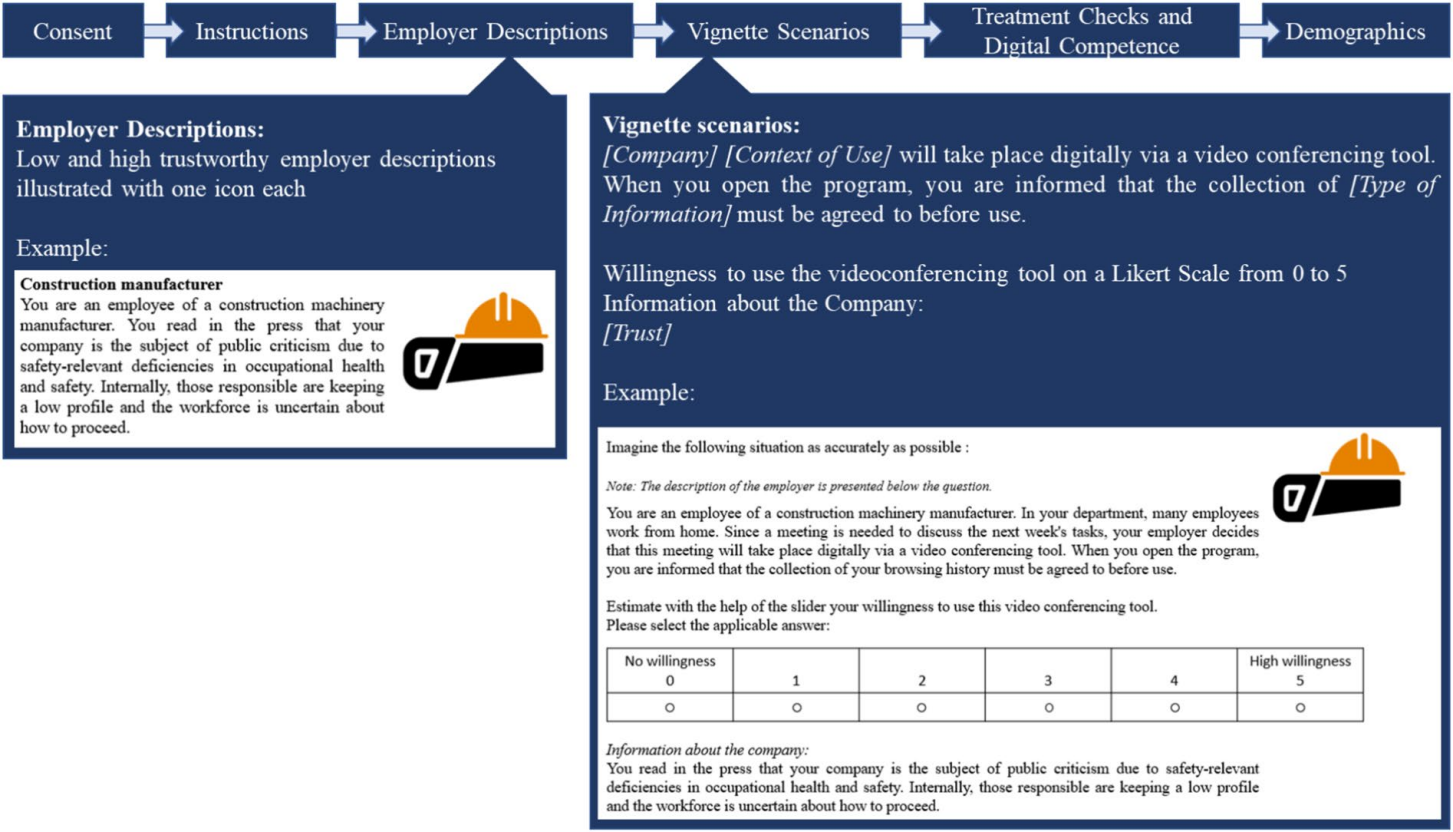
Study procedure. The figure shows the study procedure in the following order: Consent, Instructions, Employer Descriptions, Vignette Scenarios, Treatment Checks and Digital Competence and Demographics. For the employer descriptions, it is further displayed that these varied for low and high trustworthiness employer descriptions, each illustrated with an icon. Then, the employer description of the construction manufacturer (low trustworthiness employer) is shown with a helmet icon and following text:** “**You are an employee of a construction machinery manufacturer. You read in the press that your company is the subject of public criticism due to safety-relevant deficiencies in occupational health and safety. Internally, those responsible are keeping a low profile and the workforce is uncertain about how to proceed.” For vignette scenarios, the structure of each scenario is displayed with one example for the scenario ‘low trustworthiness work context of use and high sensitivity information’ shared with the following text: “Imagine the following situation as accurately as possible: Note: The description of the employer is presented below the question—You are an employee of a construction machinery manufacturer. In your department, many employees work from home offices. Since a meeting is needed to discuss the next week’s tasks, your employer decides that this meeting will take place digitally via a video conferencing tool. When you open the program, you are informed that the collection of your browsing history must be agreed to before use. Estimate with the help of the slider your willingness to use this video conferencing tool. Please select the applicable answer: [Likert Scale from 1 (no willingness) to 5 (high willingness)] *Information about the company:* You read in the press that your company is the subject of public criticism due to safety-relevant deficiencies in occupational health and safety. Internally, those responsible are keeping a low profile and the workforce is uncertain about how to proceed.”

Before the online vignette study started, each participant gave informed consent and agreed to the privacy policy. Participants were then given an instruction that asked them to put themselves in personal and work scenarios when deciding whether to use the video conferencing tool described. Subsequently, the text-based descriptions for the employers in the work scenarios were presented, which consisted of two low and two high trustworthiness employer descriptions, each illustrated with an icon (see Supplementary Material S1). This was followed by 18 text-based vignette scenarios. Each scenario described situations in which a video conferencing tool was used either in a personal context of use with friends and family or in a work context of use with the above-mentioned (un-)trustworthy employers. Additionally, the videoconferencing tool in each scenario requested one of three types of sensitive information (low, medium, high sensitivity). After rating their willingness to use the videoconferencing tool for all 18 vignette scenarios, the participants were asked to rate their trust in each described employer as well as their perceived data responsibility in personal and work contexts of use in general. Then, they were asked to state reasons for their decision to (not) use the described video conferencing tool in the given scenarios. Finally, digital competence, age, work-related data (occupation, work field, work experience, weekly working hours) as well as personal and work media use per week were collected. The study took 15–20 min to complete. The study procedure is illustrated in Fig. 1.

### Employer descriptions and vignette scenarios

The vignette scenarios varied in their contexts of use (personal and work), and within the work contexts of use they further varied in trustworthiness (low and high). As research indicates that the brand of an online provider can serve as a trust cue^36–38^, the videoconferencing tool was described without any name or brand to minimize unsystematic effects of trust in the videoconferencing tool (see Fig. 1). We used the characteristics of trustworthy companies from Böhle and co-authors^31^ (see Table 1) as a guide for varying the trustworthiness of the employer. Examples of the low and high trustworthiness employer descriptions are shown in Table 1. To improve recognition of employer descriptions, we created icons, for example, a construction helmet for the employer description of a construction company (see Supplementary Material S1). The vignettes of each condition were reformulated to reduce monotony and to prevent the recognition of single factors by participants. A *t*-test for paired samples was conducted that revealed no significant differences between the reformulations (all *p* > 0.49). We combined the response for the two reformulations for the subsequent analysis by averaging.

**Table 1:** Translated (German to English) examples of employer descriptions regarding characteristics of trustworthy companies^31^.

Characteristics of trustworthy companies^31^	Examples of (un-)trustworthy employer descriptions
Employee integration into the ongoing work process	“The problems affect you directly and you have already tried to talk to your boss several times, but he always refers you to the human resources department.” (low trustworthiness)
Personnel responsibility	“You read in the press that your company is the subject of public criticism due to safety-relevant deficiencies in occupational health and safety.” (low trustworthiness)
Mutual support in self-coordination	“You have a good relationship with your colleagues and much freedom to decide, at the same time your work is recognized and valued. Your supervisor is your mentor and supports you.” (trustworthy)
Employee participation in reorganization or optimization	“Recently, there have again been problems with the communication of internal processes” (low trustworthiness) “Internally, those responsible are keeping a low profile (low trustworthiness)

The table shows translated examples of employer descriptions regarding characteristics of trustworthy companies by Böhle et al.^31^ as described in the following: *Employee integration into the ongoing work process*, “The problems affect you directly and you have already tried to talk to your boss several times, but he always refers you to the human resources department.” (low trustworthiness); *Personnel responsibility*, “You read in the press that your company is the subject of public criticism due to safety-relevant deficiencies in occupational health and safety.” (low trustworthiness); *Mutual support in self-coordination*, “You have a good relationship with your colleagues and much freedom to decide, at the same time your work is recognized and valued. Your supervisor is your mentor and supports you.” (trustworthy); *Employee participation in reorganization or optimization*, “Recently, there have again been problems with the communication of internal processes” (low trustworthiness), “Internally, those responsible are keeping a low profile (low trustworthiness).

Moreover, we systematically varied the type of information shared with the videoconferencing tools in terms of its sensitivity: low (demographic data), medium (location data) and high (browsing history). The sensitivity of information was based on similar differentiations in a vignette study from Martin^42^. Further, it was verified through pre-tests, which showed significant differences for all three types of information sensitivity in the planned direction: the willingness to use a videoconferencing tool was highest for low sensitivity and lowest for high sensitivity. Table 2 shows the text-based vignette scenarios divided into context of use, employer trustworthiness and type (sensitivity) of information, along with its reformulation.

**Table 2:** Translated (from German to English) text-based scenarios of the vignettes divided into the vignette dimensions.

Vignette dimension	Dimension level	Operationalized in vignette
Context of use	Personal	Your former school friends now live all over Germany. Since you see them so rarely, you have decided together that the next game night …
		*or* Your next family reunion is coming up. Since your family does not share a common residence, you have decided together that the next family reunion …
	Work	Many employees are currently working in the field. Since a joint departmental meeting is scheduled, your employer decides that this meeting …
		*or* In your department, many employees work from home offices. Since a meeting is needed to discuss the next week’s tasks, your employer decides that this meeting …
Trustworthiness in work contexts of use	Low	You are an employee of a construction machinery manufacturer. You read in the press that your company is the subject of public criticism due to safety-relevant deficiencies in occupational health and safety. Internally, those responsible are keeping a low profile and the workforce is uncertain about how to proceed
		*or* You are an employee of a personnel service provider. Recently, there have again been problems with the communication of internal processes and with the payment of overtime. The problems affect you directly and you have already tried to talk to your boss several times, but he always refers you to the human resources department
	High	You are an employee of a food manufacturer. The company has been operating regionally for 100 years and is family-owned. After successfully completing your training, you are now starting a permanent position. It is foreseeable that you will advance in the company in the medium to long term
		*or* You are an employee of an automotive supplier company. The company is known for its employee-friendly conditions and fair compensation. You have a good relationship with your colleagues and much freedom to decide, at the same time your work is recognized and valued. Your supervisor is your mentor and supports you
Type of information	Low Sensitivity	Demographic data (age, gender, birthday)
	Medium Sensitivity	Location data
	High Sensitivity	Browsing history

The table shows the vignette text-based scenarios for the vignette dimensions context of use (personal, work), trust (low, high) and type of information (low, medium, high), which are additionally represented in Supplementary Material S2 for accessibility.

### Post-hoc questionnaires

In Supplementary Material S2, all questionnaires are available in German (original language) and English (translated).

#### Treatment checks

We queried trust in the employer with three items of Joinson and co-authors^33^ on the trust dimensions benevolence, integrity, and overall trust^30,33,54^ on a Likert scale from 1 (I do not agree at all) to 5 (I fully agree). Additionally, perceived responsibility for data protection in work and personal contexts of use through one self-created item was queried on a Likert scale from 1 (I do not agree at all) to 5 (I fully agree) and the reasons for (not) using the videoconferencing tool were queried through an open-ended question.

#### Digital competence

A questionnaire by Rubach and Lazarides^50^ was used to determine the participant’s self-assessed digital competence in the digital competence areas (1) seek & process, (2) communicate & collaborate, (3) protect & act safely, (4) problem-solving & act, as well as (5) analyze & reflect. The area (6) produce & present was not included due to its focus on the development of digital content^49,51^. A total of 16 items were rated on a Likert scale from 1 (I do not agree at all) to 5 (I totally agree). Reliability analysis showed a sufficient to satisfying internal consistency for all five subscales (Cronbach’s *ɑ* ranging from 0.64 to 0.81) resulting in an overall good internal consistency of *ɑ* = 0.81.

#### Demographics

Age, gender, work-related data (main occupation, work field, work experience, weekly working hours) and media use per week in personal and work contexts of use were queried.

### Participants

A total of 93 participants (*M* = 27.1 years, *SD* = 7.9) answered the complete vignette study and questionnaire. Demographic information is summarized in Table 3. Participants indicated an average self-assessed digital competence score of 4.0 (on a Likert scale from 1 to 5). Table 4 shows means and standard deviation for each digital competence area. Participants were recruited via email or social media (e.g., LinkedIn) and agreed to data collection before participation. Participation was without any payment or reward.

**Table 3:** Participants’ demographic information.

Demographic information	N = 93
Age	18 to 61 years, *M* = 27.1 years, *SD* = 7.9
Gender	46.2% male
	48.4% female
	1.1% non-binary
	4.3% not indicated
Average working time	*M* = 26.8 h per week
Average work experience	*M* = 5.4 years
Main occupation	51.6% student
	35.5% full-time
	4.3% part-time
	3.2% self-employed
Main work field	19.4% IT (Information Technology), Computer
Time spent with media use (e.g. computer, smartphone)	51.6% less than 10 h per week (leisure time)
	45.2% more than 10 h per week (leisure time)
	23.7% less than 10 h per week (work time)
	73.1% more than 10 h per week (work time)
	3.2% not indicated

The table shows the demographic information of 93 participants ranging in age from 18 to 61 years with a mean age of 27.1 (*SD* = 7.9). 46.2% of the participants were male, while 48.4% were female. In addition, 1.1% were non-binary and 4.3% did not indicate their gender. Participants reported an average working time of 26.8 h per week with an average work experience of 5.4 years, and most (19.4%) reported IT or computing as their work area. Most participants were students (51.6%), followed by full-time (35.5%), part-time (4.3%), and self-employed (3.2%). In their leisure time, most participants (51.6%) reported spending less than 10 h per week using digital media (e.g., computer, smartphone), while in their work time, most participants (73.1%) reported spending more than 10 h per week using digital media. 3.2% of participants did not report their time spent with media per week.

**Table 4:** Means (M) and standard deviation (SD) for digital competence items.

Digital competence area	Item example	*M*	*SD*
(1) Seek & process	I can identify and use relevant sources in digital environments	4.24	0.63
(2) Communicate & collaborate	I choose digital media for targeted communication opportunities according to the situation	4.46	0.55
(3) Protect & act safely	I know the dangers and risks in digital environments, consider and reflect on them	3.49	0.76
(4) Problem solving & act	I can adapt digital environments and tools for personal use	3.80	0.74
(5) Analyze & reflect	I can analyze the effect of media in the digital world and deal with it constructively	4.05	0.71

The table shows the means and standard deviation for digital competence items in five digital competence areas: (1) Seek & process, *Mean* = 4.24, *Standard Deviation* = 0.63; (2) Communicate & collaborate, *Mean* = 4.46, *Standard Deviation* = 0.55; (3) Protect & act safely: *Mean* = 3.49, *Standard Deviation* = 0.76; (4) Problem solving & act: *Mean* = 3.80, *Standard Deviation* = 0.74; (5) Analyze & reflect: *Mean* = 4.05, *Standard Deviation* = 0.71.

#### Ethics

All participants gave written informed consent in accordance with the Declaration of Helsinki^55^ and participation was voluntary. Further, no undue physical or psychological stress was anticipated. Participants were fully informed about the aims and procedures of the study and could withdraw their participation at any time. No ethical concerns in accordance with the ethics guidelines of the DFG—Deutsche Forschungsgemeinschaft (German Research Foundation) were identified^56^ and therefore no further evaluation by an ethics committee was sought.

#### Sample size

The sample size was calculated a-priori with GPower^57^. In accordance with our 3 × 3 within-subjects design, we calculated an a-priori power analysis for a repeated measures ANOVA with the following parameters: (1) effect size: *f* = 0.25, (2) alpha level:* α* = 0.05, (3) power: 0.95, (4) number of groups: 2, (5) number of measurements: 3, (6) Correlation between repeated measures: 0.5, (7) correction for non-sphericity: ε = 1. This resulted in a minimum sample size of 44 participants. In order to increase the informational value of our study, we decided to collect at least 44 participants and maximize the sample size within the study period. This resulted in a total of 93 participants at the end of the study period.

## Results

Data can be retrieved from 10.18154/RWTH-2022-05069.

### Trust in the employer: treatment check

To test whether (un)trustworthy employer descriptions decreased and increased participants’ perceived trust in the employer in the perceived direction, a repeated measures *t*-test was calculated. The *t*-test showed that trust in the employer is significantly higher in the trustworthy condition (*M* = 3.86, *SD* = 0.72) compared to the low trustworthiness condition (*M* = 2.22, *SD* = 0.68), *t* (92) = 13.52, *p* < 0.001, indicating that the manipulation of trustworthiness was successful.

### Context of use, type of information, digital competence

To examine the impact of *(1) context of use*, *(2) type of information* and *(3) self-assessed digital competence,* a 3 × 3 repeated measures ANCOVA with the within-subject factors *context of use* (personal vs. high trustworthiness work vs. low trustworthiness work) and *type of information* (low, medium, high sensitivity) was calculated. Mean scores of digital competence (*z*-standardized) served as the covariate. Assumptions were checked and the willingness to use the videoconferencing tool and digital competence were approximately normally distributed. Violations of sphericity were adjusted with Huynh-Feld. We report ${\eta }_{p}^{2}$ as a measure of effect size^58^.

The data revealed a significant effect of *context of use* with *F* (2, 182) = 45.354, *p* < 0.001, ${\eta }_{p}^{2}$ = 0.333. Participants’ willingness to use the videoconferencing tool was highest in the high trustworthiness work context of use (*M* = 3.78, *SD* = 0.10), followed by the personal context of use (*M* = 3.67, *SD* = 0.11) and lowest in the low trustworthiness work context of use (*M* = 3.08, *SD* = 0.10). Bonferroni-adjusted post-hoc tests showed that the low trustworthiness work context of use significantly differs from the high trustworthiness work context of use (*p* < 0.001), as assumed in Hypothesis 1. However, no significant difference between the high trustworthiness work context of use and the personal context of use (*p* = 0.454), as expected in Hypothesis 2, were revealed. An overview of all Bonferroni-adjusted post-hoc comparisons is shown in Table 5.

**Table 5:** Bonferroni-adjusted post-hoc analysis for type of information and context of use.

Main effect	Compared factor level	Mean difference	*p* value
Type of information	low vs. medium sensitivity	0.20	*p* = 0.395
Type of information	low vs. high sensitivity	1.84	*p* < 0.001
Type of information	medium vs. high sensitivity	1.65	*p* < 0.001
Context of use	personal vs. high trustworthiness work	-0.11	*p* = 0.454
Context of use	personal vs. low trustworthiness work	0.59	*p* < 0.001
Context of use	high vs. low trustworthiness work	0.70	*p* < 0.001

Mean differences were calculated by subtracting the former from the latter mean for each factor level.

The table shows the results from the Bonferroni-adjusted post-hoc analysis with the following mean differences and *p*-values: (1) Type of Information for low vs. medium: Mean difference = 0.20, *p* = 0.395, for low vs. high: Mean difference = 1.84, *p* < 0.001 and for medium vs. high: Mean difference = 1.65, *p* < 0.001 and (2) Context of use for personal vs. trust-work Mean difference = -0.11, *p* = 0.454, for personal vs. non-trust work: Mean difference = 0.59, *p* < 0.001 and for trust work vs. non-trust work Mean difference = 0.70, *p* < 0.001.

Our analysis revealed a significant main effect for *type of information*, *F* (1.812, 164.930) = 84.948, *p* < 0.001, ${\eta }_{p}^{2}$ = 0.483. Participants’ willingness to use the videoconferencing tool was highest for low sensitivity information types of shared data (*M* = 4.19, *SD* = 0.14), followed by medium sensitivity (*M* = 3.99, *SD* = 0.12), and lowest for high sensitivity information types of shared data (*M* = 2.35, *SD* = 0.13). Bonferroni-adjusted post-hoc tests showed that participants were significantly less willing to use the video conferencing tool when sharing was requested for data of high sensitivity than when medium or low sensitivity information was requested (*p* < 0.001, see Table 5). This finding is in line with Hypothesis 3.

**Fig. 2:**
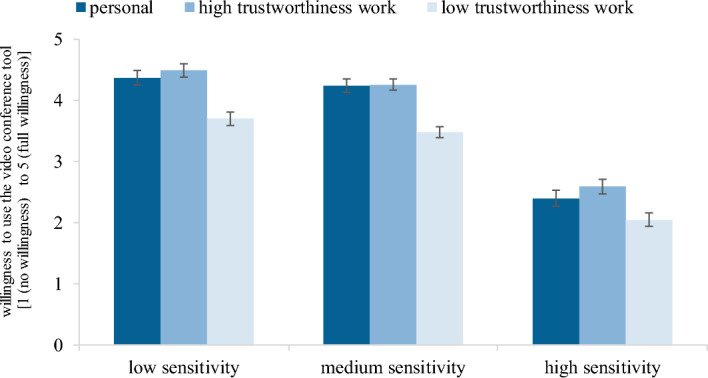
Mean willingness to use the videoconferencing tool [0 (no willingness) to 5 (full willing-ness)] for context of use and type of information (N = 93). Note: Error bars indicate within-subject standard errors of the means^59^. The figure shows nine bar graphs. The x-scale shows the willingness to use the videoconference tool from 1 (no willingness) to 5 (high willingness). The y-scale shows low, medium and high sensitivity of shared information divided for personal, work-high trust and work-low trust contexts of use. The following mean willingness is presented: 4.37 for low sensitivity and personal, 4.49 for low sensitivity and work-high trust, 3.70 for low sensitivity and work-low trust; 4.24 for medium sensitivity and personal, 4.26 for medium sensitivity and work-high trust, 3.48 for medium sensitivity and work-low trust; 2.40 for high sensitivity and personal, 2.59 for high sensitivity and work-high trust, 2.05 for high sensitivity and work-low trust.

Moreover, we observed a significant interaction between *context of use* and *type of information* with *F* (3.658, 332.856) = 2.761, *p* = 0.032, ${\eta }_{p}^{2}$ = 0.029. The difference between personal contexts of use or high trustworthiness contexts on the one hand and low trustworthiness work contexts of use on the other is reduced where the *type of information* is high sensitivity compared to low or medium sensitivity (Fig. 2). The covariate *digital competence* had no significant influence on the overall willingness to use the videoconferencing tool (*p* = 0.895) and did not significantly impact the *context of use* effect (*p* = 0.250), as supposed in Hypothesis 4.1 and 4.2. No other significant effects were observed (all *p* values > 0.21).

### Perceived responsibility

Because users might shift the responsibility for their data protection to trustworthy employers in work contexts of use, we expected the willingness to use will also be higher in trustworthy work contexts of use than in personal contexts of use (Hypothesis 2). To examine shifted responsibility in work compared to personal contexts of use, a two-sided *t*-test for dependent samples was conducted. A significant difference between work (*M* = 3.01, *SD* = 1.14) and personal context (*M* = 4.60, *SD* = 0.77) was found, *t* (92) = -11.48, *p* < 0.001, indicating that participants felt more responsible for the protection of their data in their personal context than in their work context. This confirms the responsibility shift we had assumed.

### Open answers

To gain insight into the reasons for participants’ willingness to use or not use the videoconferencing tool, open answers were coded by one author (see Table 6). It should be noted that open-ended answers were optional and 36 participants (38.71%) provided open answers with an average of about 40 words per answer (*M* = 40.08, *SD* = 45.34). Therefore, these should be interpreted with caution. Open answers revealed that the willingness of participants to use the videoconferencing tool was often due to being able to understand the sense and purpose for the type of personal data recorded (11) or depended on the context for which data is used (7). Furthermore, some participants indicated that they reject data sharing in general (6) or that they felt like they had no other choice (6).

**Table 6:** Coding categories, frequency, and examples.

Coding category	Examples	Frequency in total numbers
Sense, purpose, relevance, or transparency in data release	“I am almost used to disclosing demographic data and find it quite ‘general’ and therefore justifiable.”	11
Context dependent	“It depends on the situation, both privately and professionally.”	7
Having no choice, peer pressure	“If it is in a work context, you do not have the opportunity to refuse because e.g., a warning threatens.”	6
General rejection	“The browser history is nobody’s business, regardless of whether the company is good or bad.”	6
Indication for omission bias	“If my employer thinks that this data is not in need of protection, I accept the decision.”	3
Trust in the employer	“Bad working relationships lead to less willingness.”	2
Trust for the tool	“Trustworthiness of the tool, technologies used, encryption.”	2
Avoiding problem by deliberately giving false information	“I have no concerns about querying demographic data – I just give the wrong ones.”	1
Not assigned to a category	“Browsing history priority no”	1

The table shows the coding categories, frequency, and examples with the following coding categories and frequencies in total numbers: (1) Sense, purpose, relevance, or transparency in data release, *N* = 11; (2) Context dependent, *N* = 7; (3) Having no choice, peer pressure, *N* = 6; (4) General rejection, *N* = 6; (5) Indication for omission bias, *N* = 3; (6) Trust in the employer, *N* = 2; (7) Trust for the tool, *N* = 2; (8) Avoiding problem by deliberately giving false information, *N* = 1; (09) Not assigned to a category, *N* = 1.

## Discussion

The study purpose was to understand how employer trustworthiness and potential shifts in responsibility in work contexts of use affect the willingness to use third party video conferencing tools. As expected, the results indicate employer trustworthiness as a pivotal factor in determining the willingness to use third-party video conferencing tools. In contrast, differences between personal and work contexts of use, characterized by differences in perceived responsibility for data protection, do not necessarily determine the willingness to use this type of videoconferencing tool. In addition, our results indicate that users’ willingness to use a videoconferencing tool is influenced by the sensitivity of the type of information it requires to be shared, but not by their self-assessed level of digital competence.

### Employer’s trustworthiness

Similarly, to the strong influence of trust indicated in the privacy literature^10,16,28^, we found that users’ willingness to use video conferencing tools seemed to depend on the trustworthiness of the employer. More precisely, participants were more willing to use a videoconferencing tool, and accept its privacy policy, if they trusted the employer who recommended the tool.

This finding provides new insights into users’ privacy behavior because users were influenced by the trustworthiness of an independent person such as the employer and not the data recipient, i.e. the operator of the videoconferencing tool. This raises the question of why users would share their personal information by using the video conferencing tool based on the trustworthiness of someone who is not responsible for handling their data. One explanation, which has already been mentioned above, could be the users’ tendency to rely on certain short-cuts, so-called heuristics, in online privacy decision-making^8,9^. Some researchers identified that trust can be used as an anchor, as it influences the subsequent assessment of ambiguous and uncertain situations and reduces cognitive load^40^. An *anchor heuristic* is characterized by a person’s tendency to “anchor their initial estimation of information that readily comes to mind, and then adjust their thinking around that anchor in what seems like an appropriate direction” (p.19)^40^. Such trust anchoring effects may have influenced the participants’ privacy decisions as the vignette scenarios contained little information about where data (e.g., demographic data, location, browser history) is stored, by whom (e.g., known company or brand) and how it is processed. Consequently, participants may have used employer trustworthiness as an anchor to reduce the uncertainty of their privacy decisions. Alternatively, information overload due to limited mental resources (cf. bounded rationality^8,9^) could serve as an explanation. Participants might use employer trustworthiness as an anchor to reduce the cognitive load associated with privacy decisions at work^8,40^. To extend our findings, as an alternative explanation approach, future research could investigate whether the level of trust in the employer might impact the users’ feeling of responsibility for their data protection.

### Work and personal contexts of use: differences in perceived responsibility

We also assumed that users’ willingness to use a videoconferencing tool and accept its privacy policies would be higher for the trustworthy work context than for the personal context of use, since users might feel less responsible for their data protection in a trustworthy work context of use. However, even if our treatment check revealed the expected differences in perceived responsibility, with higher felt responsibility in the personal context than in work contexts, we did not find differences in users’ willingness to use a videoconferencing tool between those contexts of use. As the personal contexts of use which we investigated were situated within social scenarios (e.g., meetings with family or friends), social influence could be one explanation for this finding. The impact of the felt shift in responsibility might be reduced or less visible where users trusted the recommendation of friends and family for the respective tool (social cueing^35^), or through *Fear of Missing Out* (FoMO)^60,61^ on the family reunion and game evening with friends. To further investigate differences in context of use, future research should control for social effects (e.g. trust in friends or FoMO). One approach could be to include low trustworthiness personal contexts of use (e.g., with an unreliable and tardy friend) and use these in comparisons. Another explanation may lie in the privacy-related dynamics of online services, as outlined by Masur^6^. The social scenarios may have created a sense of privacy among users (horizontal level) as information was shared within in a family reunion or game evening with friends. This sense of privacy in the personal context of use may have blurred the users’ assessment of the privacy risks between them and the company operating the videoconferencing tool (vertical level)^6^.

### Type of sensitive information

The scenarios were varied for three types of shared information (low, medium, high sensitivity), because sharing of different types of sensitive information is known as an important contextual characteristic^4^ and may be perceived differently^25,42^. Our data confirmed a higher willingness to disclose low or medium sensitivity information compared to high sensitivity. However, the results did not a show higher willingness to disclose demographic (low sensitivity) compared to location data (medium sensitivity) as suggested by previous studies^42,44^. These findings may indicate a relation to perceived functional relevance of the data: demographic and location data might be considered functionally relevant to a video conferencing tool, while browser history was not. The open-ended answers confirmed this type of tendency, because participants identified the purpose and context for which data was used as an important criterion for their willingness to use the described video conferencing tool. Privacy appears to be contextual, with users considering the sensitivity of data in relation to its context. This corresponds with previous research, where researchers have found that sharing certain types of information can be perceived as more or less risky depending on the context. For example, health information (e.g., number and kind of diseases) was more willingly shared in a medical context (relevant) compared to retail (irrelevant) contexts^25^. Another potential explanation may lie in the granularity of the data, particularly location data. Online services often request users to provide information about their location, but according to Martin and Nissenbaum^43^, the location request may involve different levels of sensitivity and thus differentially affect perceptions of appropriateness^43^. As a result, the location data in our scenarios may have been perceived as less sensitive, as it was considered on a broader level (e.g., city) by the participants.

In addition, the type of sensitive information seemed to influence the trust effect: differences in users’ willingness to use a video conferencing tool diminished for highly sensitive types of information such as browsing history. This may indicate that a high degree of information sensitivity leads users to make conscious privacy decisions independent of employer trustworthiness. However, more research is needed to determine under what circumstances employer trustworthiness increases or decreases the willingness of users to share personal information. Future research could investigate whether trust effects diminish for other types of sensitive data (e.g., phone number^44^).

### Digital competence

To identify whether digital competence is associated with users’ willingness to use the video conferencing tool and the associated privacy decision, we queried participants’ digital competence. Contrary to our initial assumption, for self-assessed digital competence, we found neither an impact on users’ willingness to use the video conferencing tool (Hypothesis 4.1) nor an impact on the expected difference between personal and work contexts of use (Hypothesis 4.2). As previous research has found that general Internet skills^48^ or generic technical familiarity^47^ affect users’ privacy control behavior, it is important to closely examine our sample and measurement of digital competence and its relation to privacy behavior. First, as our random sample was rather young and many indicated they work in fields such as IT/computer or academic studies, there were high scores of *digital competences.* Thus, it can be argued that we did not measure the impact of digital competence because our sample did not differentiate well enough (ceiling effect). However, our findings may also indicate difficulties in recording self-assessment of digital competence by means of the questionnaire from Rubach and Lazarides^50^ used in this study. Users’ self-assessment of digital competence is one common approach; however, it is criticized for integrating users’ perception of digital competence^62^. It is unclear whether self-assessed digital competence can be equated with actual digital competence^50^ so the lack of a correlation between users’ privacy decisions and measured digital competence may be due to not capturing actual digital competence. More research is needed to enhance the measurement of digital competence, e.g., through a universal assessment instrument^62^.

### Practical implications

Our findings underscore how users’ willingness to share personal information online is influenced by their trust in their employers, who often cannot affect the data processing of recommended remote work options such as Zoom, MS Teams, or Skype. Thus, our findings suggest that a trustworthy relationship between employers and employees is likely to be critical to the adoption of remote work options. Employers who are struggling to establish remote work options in their workforce should therefore consider how they can increase general employee trust in order to increase compliance. However, employers must also be mindful of their responsibility to protect employees’ personal information while working, as users appear to be less protective of their personal information when the employer is trusted. This could expose employees and their personal data to privacy risks. Rather than just protecting company data, employers should ensure the use of privacy-friendly remote working options and inform their employees about their associated privacy risks during working hours. According to Bhave and co-authors^64^, emerging technological trends in the workplace, such as wearable devices, robots, or biometrics, further underscore the need for employers to develop appropriate consent procedures about the data collected and the risks involved^64^. Furthermore, as our results did not show the hypothesized influence of digital competence, it is important to identify whether or not digital competence training in future educational efforts can improve self-determined and deliberated privacy decisions at work. Considering the importance of privacy literacy on users’ privacy protection behavior highlighted in other studies^45,46^, future research could also investigate how to improve privacy literacy in the workplace.

### Methodological limitations

It is important to consider the limitations of vignette survey methodology when interpreting our findings. The experimental online vignette methodology allowed us to gain a more realistic insight into participants’ privacy decisions than traditional questionnaires^63^. However, our findings capture intended privacy behavior and are not completely generalizable to actual privacy behavior. For example, users may be influenced by other context-specific antecedents, such as the visualization of the privacy declaration. Future research should therefore investigate the contextual effects of privacy decisions with multi-method approaches, such as combining traditional survey methods with logging and experience sampling methods as highlighted by Masur^6^. Additionally, the external validity of our findings could be strengthened through field studies or qualitative approaches such as focus groups. This could help to gain deeper insights into trust and shift responsibility to the employer in the workplace. As mentioned above, it is also important to consider that our study measured digital competence through self-assessment. Even though the subscales are based on a validated questionnaire by Rubach and Lazarides^50^, it is unclear whether self-assessed digital competence can be equated with actual digital competence^50^. Therefore, the concept of digital competence and other related broader literacy concepts should be investigated further in conjunction with knowledge-based tests or actual assessments of digital competence areas.

## Conclusion

The present study revealed novel insights into users’ privacy decisions at work. Users’ willingness to use a videoconferencing tool and disclose the requested personal information increased with increased employer trustworthiness, but decreased when highly sensitive types of information were requested. Our investigation highlights the complexity of online privacy decisions and how context-dependent variables such as trust, responsibility, or information sensitivity should be investigated in future research. Additionally, the study underscores the need for more research on the concept of digital competence—this could be one strategy to increase users’ self-determined handling of data.

### Supplementary Information

Supplementary Information 1.

Supplementary Information 2.

## Data Availability

The data is available at RWTH Publications: https://doi.org/10.18154/RWTH-2022-05069.
