# Supplementary material for: How users make online privacy decisions in work and personal contexts of use
Source: Sci Rep. 2024 Aug 27;14:19849. doi: 10.1038/s41598-024-70718-7 (PMC11349749; doi:10.1038/s41598-024-70718-7)
Supplement: Supplementary file 1 — Supplementary Information 1. [file 41598_2024_70718_MOESM1_ESM.pdf]

Personal Context of Use - Your former school friends now live all over Germany. Since you see them so rarely, you have decided together that the next game night …

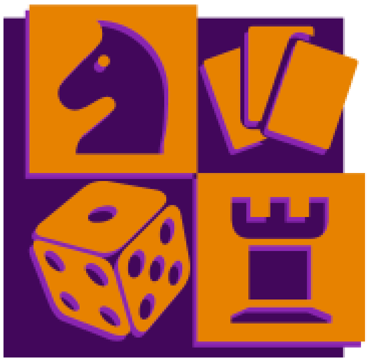

Personal Context of Use - Your next family reunion is coming up. Since your family does not share a common residence, you have decided together that the next family reunion ...

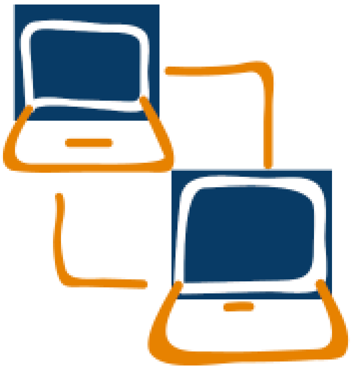

High Trust Work Context of Use - You are an employee of an automotive supplier company…

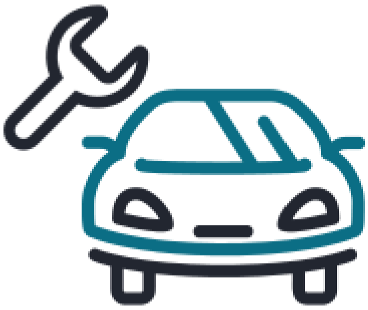

High Trust Work Context of Use - You are an employee of a food manufacturer…

Low Trust Work Context of Use - You are an employee of a personnel service provider…

Low Trust Work Context of Use - You are an employee of a construction machinery manufacturer…
