# Supplementary material for: How users make online privacy decisions in work and personal contexts of use
Source: Sci Rep. 2024 Aug 27;14:19849. doi: 10.1038/s41598-024-70718-7 (PMC11349749; doi:10.1038/s41598-024-70718-7)
Supplement: Supplementary file 2 — Supplementary Information 2. [file 41598_2024_70718_MOESM2_ESM.pdf]

Supplementary follow-up questionnaire – S2

(in the original language)

Treatment check Vertrauen (cf. Joinson et al., 2010; α = 0.91 – 0.94)

Likert Skala: 1 (stimme gar nicht zu) bis 5 (stimme voll und ganz zu)

1. Das Unternehmen ist ehrlich mit seinen Mitarbeitern.
2. Die Intentionen des Unternehmens sind gut.
3. Insgesamt ist das Unternehmen vertrauenswürdig.

Treatment check Verantwortlichkeit

Likert Skala: 1 (stimme gar nicht zu) bis 5 (stimme voll und ganz zu)

4. Im Arbeitskontext trage ich die Verantwortung für den Schutz meiner Daten.
5. Im Privatkontext trage ich die Verantwortung für den Schutz meiner Daten.

Offene Fragen:

6. Gab es weitere Gründe für ihre Entscheidung Daten freizugeben/ nicht freizugeben? Wenn ja, welche? (optional)

Digitale Kompetenz (Rubach & Lazarides, 2019; α = 0.81)

Likert Skala: 1 (stimme gar nicht zu) bis 5 (stimme voll und ganz zu)

7. Ich kann relevante Quellen in digitalen Umgebungen identifizieren und nutzen.
8. Ich kann Informationen und Daten analysieren, interpretieren und kritisch bewerten.
9. Ich kann Informationen an verschiedenen Orten speichern und diese abrufen.
10. Ich wähle digitale Medien für gezielte Kommunikationsmöglichkeiten situationsgerecht aus.
11. Ich kann Informationen, Dateien und Links teilen.
12. Ich kann digitale Medien nutzen, um gemeinsam Dateien und Dokumente zu bearbeiten.
13. Ich kann mithilfe digitaler Medien aktiv an der Gesellschaft teilhaben.
14. Ich kenne die Gefahren und Risiken in digitalen Umgebungen, berücksichtige und reflektiere diese.
15. Ich kann meine Privatsphäre in digitalen Umgebungen durch geeignete Maßnahmen schützen.
16. Ich kann digitale Technologien gesundheits- und umweltbewusst nutzen.
17. Ich kann digitale Umgebungen und Werkzeuge zum persönlichen Gebrauch anpassen.
18. Ich kenne meine Defizite bei der Nutzung digitaler Werkzeuge und kann Strategien zur Beseitigung entwickeln.
19. Ich kenne eine Vielzahl digitaler Werkzeuge und kann diese bedarfsgerecht einsetzen.
20. Ich kann die Wirkung von Medien in der digitalen Welt analysieren und konstruktiv damit umgehen.
21. Ich kenne die Vielfalt der digitalen Medienlandschaft.
22. Ich erkenne Chancen und Risiken des Mediengebrauchs und kann diese reflektieren.

Demographie

23. Bitte geben Sie Ihr Geschlecht an [Einfach-Antwort, optional: weiblich, männlich, divers]
24. Bitte geben Sie Ihr Alter an [offene Antwort, optional: in Jahren]

Arbeitstätigkeit

25. Welcher Haupttätigkeit gehen Sie derzeit nach? [Einfach-Antwort, optional: Vollzeit angestellt, Teilzeit angestellt, Selbstständig, Studium]
26. Welche Kategorie würde Ihre derzeitige Arbeitstätigkeit am besten beschreiben? [Einfach-Antwort, optional: Bau, Architektur, Vermessung; Dienstleistung; Elektro; Forschung; Gesundheit; IT, Computer; Kunst, Kultur, Gestaltung; Landwirtschaft, Natur, Umwelt; Medien; Metall, Maschinenbau; Naturwissenschaften; Produktion, Fertigung; Soziales, Pädagogik; Technik, Technologiefelder; Verkehr, Logistik; Wirtschaft, Verwaltung; Sonstiges: offene Antwort]
27. Wie viel Berufserfahrung haben Sie? [offene Antwort, optional: in Jahren]
28. Wie viele Stunden arbeiten Sie pro Woche beruflich? [offene Antwort, optional: in Stunden]
29. Wie viel Zeit verbringen Sie pro Woche am Arbeitsplatz mit digitalen Medien (Computer, Smartphone etc.)? [Einfach-Antwort, optional: 0, 1-10, 10-20, 20-30, 30-40, über 40 Stunden]
30. Wie viel Zeit verbringen Sie pro Woche in Ihrer Freizeit bzw. privat mit digitalen Medien (Computer, Smartphone etc.)? [Einfach-Antwort, optional: 0, 1-10, 10-20, 20-30, 30-40, über 40 Stunden]
31. Gibt es weitere Anmerkungen?

Translated from German—original language—to English

Treatment check Trust (cf. Joinson et al., 2010; α = 0.91 – 0.94)

Likert scale: 1 (do not agree at all) to 5 (agree completely)

1. The company is honest with its employees.
2. The company's intentions are good.
3. Overall, the company is trustworthy.

Treatment check Responsibility

Likert scale: 1 (do not agree at all) to 5 (agree completely)

4. In a work context, I am responsible for the protection of my data.
5. In a personal context, I am responsible for the protection of my data.

Open questions:

6. Were there any other reasons for your decision to release/not to release data? If yes, what were they? (optional)

Digital competence (Rubach & Lazarides, 2019; α = 0.81)

Likert scale: 1 (do not agree at all) to 5 (agree completely)

7. I can identify and use relevant sources in digital environments.
8. I can analyze, interpret, and critically evaluate information and data.
9. I can store information in different locations and retrieve it.
10. I choose digital media for targeted communication opportunities according to the situation.
11. I can share information, files, and links.

12. I can use digital media to collaborate on files and documents.
13. I can actively participate in society using digital media.
14. I know the dangers and risks in digital environments, consider and reflect on them.
15. I can protect my privacy in digital environments by taking appropriate measures.
16. I can use digital technologies in a health- and environmentally conscious way.
17. I can adapt digital environments and tools for personal use.
18. I know my deficits in the use of digital tools and can develop strategies to eliminate them.
19. I know a variety of digital tools and can use them as needed.
20. I can analyze the effect of media in the digital world and deal with it constructively.
21. I know the diversity of the digital media landscape.
22. I recognize opportunities and risks of media use and can reflect on them.

Demographic data

23. Please enter your gender [single answer, optional: female, male, diverse]
24. Please enter your age [open answer, optional: in years]

Work activity

25. What is your main activity at the moment? [Single answer, optional: Full-time employed, part-time employed, Self-employed, Studying]
26. Which category would best describe your current work activity? [single answer, optional: construction, architecture, surveying; service; electrical; research; health; IT, computer; art, culture, design; agriculture, nature, environment; media; metal, mechanical engineering; natural sciences; production, manufacturing; social affairs, education; technology, technology fields; transport, logistics; economics, administration; other: open answer]
27. How much work experience do you have? [open answer, optional: in years]
28. How many hours per week do you work professionally? [open answer, optional: in hours]
29. How much time do you spend per week at work with digital media (computer, smartphone, etc.)? [Single answer, optional: 0, 1-10, 10-20, 20-30, 30-40, over 40 hours]
30. How much time per week do you spend using digital media (computer, smartphone, etc.) in your free time or private life? [Single answer, optional: 0, 1-10, 10-20, 20-30, 30-40, over 40 hours]
31. Are there any other comments?

Supplementary Text-Based Vignettes - S2

Text-based vignettes in the original language (German)

[VERTRAUEN]. [NUTZUNGSKONTEXT] digital über ein Videokonferenztool stattfinden wird. Als Sie das Programm öffnen, werden Sie darüber informiert, dass der Erfassung von [INFORMATIONSTYP] vor Nutzung zugestimmt werden muss.
Niedrig: Sie sind Beschäftigte*r eines Baumaschinen-Herstellers. (kV) In der Presse lesen Sie, dass Ihr Unternehmen aufgrund sicherheitsrelevanter Mängel im Arbeitsschutz in der öffentlichen Kritik steht. Intern halten sich die Verantwortlichen bedeckt und die Belegschaft ist verunsichert, wie es weitergeht. *oder* Sie sind Beschäftigte*r eines Personaldienstleisters (kV). Jüngst gab es erneut Probleme in der Kommunikation interner Prozesse und mit der Bezahlung von Überstunden. Die Probleme betreffen Sie direkt und Sie suchten bereits mehrmals das Gespräch mit Ihrem Chef, der Sie jedoch immer an die Personalabteilung verweist.
Hoch: Sie sind Beschäftigte*r eines Lebensmittelherstellers. (V) Das Unternehmen ist seit 100 Jahren regional tätig und befindet sich in Familienhand. Nachdem Sie die Ausbildung erfolgreich abgeschlossen haben, treten Sie nun eine Festanstellung an. Es ist abzusehen, dass Sie im Unternehmen mittel- bis langfristig aufsteigen. *oder* Sie sind Beschäftigte*r einer Automobilzulieferfirma (V). Das Unternehmen ist bekannt für seine mitarbeiterfreundlichen Rahmenbedingungen und die faire Vergütung. Sie haben ein gutes Verhältnis zu Ihren Kolleg*innen und viele Entscheidungsspielräume, außerdem wird Ihre Arbeit anerkannt und wertgeschätzt. Ihre Vorgesetzte ist Ihre Mentorin und fördert Sie.

[VERTRAUEN]. [NUTZUNGSKONTEXT] digital über ein Videokonferenztool stattfinden wird. Als Sie das Programm öffnen, werden Sie darüber informiert, dass der Erfassung von [INFORMATIONSTYP] vor Nutzung zugestimmt werden muss.
Arbeit: Viele Mitarbeiter sind derzeit im Außendienst tätig. Da eine gemeinsame Besprechung der Abteilungen ansteht, entscheidet ihr Arbeitgeber, dass dieses Meeting … *oder* In ihrer Abteilung sind viele Mitarbeiter im Home-Office tätig. Da eine Besprechung der nächsten Wochenaufgaben nötig ist, entscheidet ihr Arbeitgeber, dass dieses Meeting ...
Privat: Ihre früheren Schulfreunde wohnen mittlerweile über ganz Deutschland verteilt. Da Sie diese so selten sehen, haben sie sich gemeinsam entschieden, dass der nächste Spieleabend … *oder* Ihr nächstes Familientreffen steht an. Da ihre Familie keinen gemeinsamen Wohnort teilt, haben Sie gemeinsam entschieden, dass das nächste Familientreffen ...

[VERTRAUEN]. [NUTZUNGSKONTEXT] digital über ein Videokonferenztool stattfinden wird. Als Sie das Programm öffnen, werden Sie darüber informiert, dass der Erfassung von [INFORMATIONSTYP] vor Nutzung zugestimmt werden muss.
Niedrig: demographische Daten (Alter, Geschlecht, Geburtstag)
Mittel: Standortdaten
Hoch: Browserverlauf

Text-based vignettes translated from German to English
[TRUST]. [CONTEXT OF USE] will take place digitally via a video conferencing tool. When you open the program, you are informed that the collection of [TYPE OF INFORMATION] must be agreed to before use.
Low: You are an employee of a construction machinery manufacturer. You read in the press that your company is the subject of public criticism due to safety-relevant deficiencies in occupational health

and safety. Internally, those responsible are keeping a low profile and the workforce is uncertain about how to proceed. *or* You are an employee of a personnel service provider. Recently, there have again been problems with the communication of internal processes and with the payment of overtime. The problems affect you directly and you have already tried to talk to your boss several times, but he always refers you to the human resources department.
High: You are an employee of a food manufacturer. The company has been operating regionally for 100 years and is family-owned. After successfully completing your training, you are now starting a permanent position. It is foreseeable that you will advance in the company in the medium to long term. *or* You are an employee of an automotive supplier company. The company is known for its employee-friendly conditions and fair compensation. You have a good relationship with your colleagues and much freedom to decide, at the same time your work is recognized and valued. Your supervisor is your mentor and supports you.

[TRUST]. [CONTEXT OF USE] will take place digitally via a video conferencing tool. When you open the program, you are informed that the collection of [TYPE OF INFORMATION] must be agreed to before use.
Work: Many employees are currently working in the field. Since a joint departmental meeting is scheduled, your employer decides that this meeting … *or* In your department, many employees work from home offices. Since a meeting is needed to discuss the next week's tasks, your employer decides that this meeting ...
Personal: Your former school friends now live all over Germany. Since you see them so rarely, you have decided together that the next game night … *or* Your next family reunion is coming up. Since your family does not share a common residence, you have decided together that the next family reunion ...

[TRUST]. [CONTEXT OF USE] will take place digitally via a video conferencing tool. When you open the program, you are informed that the collection of [TYPE OF INFORMATION] must be agreed to before use.
Low: demographic data (age, gender, birthday)
Medium: location data
High: browsing history
